# Quantification and Proximal-to-Distal Distribution Pattern of Tibial Nerve Lesions in Relapsing-Remitting Multiple Sclerosis: Assessment by MR Neurography

**DOI:** 10.1007/s00062-022-01219-1

**Published:** 2022-10-20

**Authors:** Adriana M. Pietsch, Andrea Viehöver, Ricarda Diem, Markus Weiler, Mirjam Korporal-Kuhnke, Brigitte Wildemann, Georges Sam, John M. Hayes, Olivia Fösleitner, Johann M. E. Jende, Sabine Heiland, Martin Bendszus, Jennifer C. Hayes

**Affiliations:** 1 Department of Neuroradiology, Heidelberg University Hospital, Im Neuenheimer Feld 400, 69120 Heidelberg, Germany; 2 Department of Internal Medicine, Spital Walenstadt, Walenstadt, Switzerland; 3 Department of Neurology, Heidelberg University Hospital, Heidelberg, Germany; 4 Department of Neurology, University of Michigan, Ann Arbor, USA; 5 Division of Experimental Radiology, Department of Neuroradiology, Heidelberg University Hospital, Heidelberg, Germany

**Keywords:** Magnetic resonance neurography, Peripheral nervous system, Quantitative imaging markers, Proton spin density, T2-relaxometry

## Abstract

**Purpose:**

Recent studies suggest an involvement of the peripheral nervous system (PNS) in multiple sclerosis (MS). Here, we characterize the proximal-to-distal distribution pattern of peripheral nerve lesions in relapsing-remitting MS (RRMS) by quantitative magnetic resonance neurography (MRN).

**Methods:**

A total of 35 patients with RRMS were prospectively included and underwent detailed neurologic and electrophysiologic examinations. Additionally, 30 age- and sex-matched healthy controls were recruited. 3T MRN with anatomical coverage from the proximal thigh down to the tibiotalar joint was conducted using dual-echo 2‑dimensional relaxometry sequences with spectral fat saturation. Quantification of PNS involvement was performed by evaluating microstructural (proton spin density (ρ), T2-relaxation time (T2_app_)), and morphometric (cross-sectional area, CSA) MRN markers in every axial slice.

**Results:**

In patients with RRMS, tibial nerve lesions at the thigh and the lower leg were characterized by a decrease in T2_app_ and an increase in ρ compared to controls (T2_app_ thigh: *p* < 0.0001, T2_app_ lower leg: *p* = 0.0040; ρ thigh: *p* < 0.0001; ρ lower leg: *p* = 0.0098). An additional increase in nerve CSA was only detectable at the thigh, while the semi-quantitative marker T2w-signal was not altered in RRMS in both locations. A slight proximal-to-distal gradient was observed for T2_app_ and T2-signal, but not for ρ.

**Conclusion:**

PNS involvement in RRMS is characterized by a decrease in T2_app_ and an increase in ρ, occurring with proximal predominance at the thigh and the lower leg. Our results indicate microstructural alterations in the extracellular matrix of peripheral nerves in RRMS and may contribute to a better understanding of the pathophysiologic relevance of PNS involvement.

## Introduction

Multiple sclerosis (MS) is traditionally regarded as an autoimmune chronic inflammatory demyelinating disease restricted to the central nervous system (CNS). The exact etiology and pathomechanism remains unclear, but a multifactorial genesis combining genetic and environmental factors, such as low vitamin D levels, is currently favored [1]. Initial clinical manifestations often affect patients’ vision due to optic neuritis or lead to sensory or motor impairments [2, 3]. Later on, further motor restrictions, autonomic dysfunction [4] and neuropsychologic symptoms can occur [5, 6]. Approximately 90% of all cases manifest as relapsing-remitting MS (RRMS), with some cases transforming into secondary progressive MS (SPMS), while the remaining 10% are considered as chronic progressive (CPMS) [7, 8]. With the latest revision of the McDonald criteria from 2017, the diagnosis of MS can be established after one single clinical event when magnetic resonance imaging (MRI) of the CNS confirms a dissemination of inflammatory lesions in space and time [9].

An involvement of the peripheral nervous system (PNS) in MS has been controversially discussed in the literature since early histopathologic studies found areas of demyelination in peripheral nerves of deceased MS patients [10–12]. Electrophysiologic studies were published with inconclusive findings neither proving nor refuting a PNS involvement [13, 14]. In a recent proof-of-concept study applying high-resolution MR neurography (MRN), PNS lesions in the sciatic nerves of MS patients were for the first time visualized in vivo [15]. In that study, nerve abnormalities were derived from alterations of the T2w signal, [15] a per se unspecific marker that cannot be directly quantified, and that might be influenced by external factors such as field inhomogeneities, or different signal attenuation for the imaged slabs [16]. However, additional nerve lesion quantification, using T2-relaxometry, was only performed at a small anatomical region at the distal thigh.

With this study, we aimed to characterize and quantify PNS involvement on a microstructural level by applying T2-relaxometry, and to identify the proximal-to-distal nerve lesion distribution pattern in a clinically and electrophysiologically well-characterized cohort of patients with RRMS in comparison with healthy controls.

## Methods

### Study Design

This cross-sectional, single center study was approved by the local ethics committee (S-405/2012) and all participants gave written informed consent according to the Declaration of Helsinki. Overall, 35 patients with RRMS (12 males, 23 females, mean age 37.7 ± 2.2 years, range 20–64 years, 2017 McDonald criteria fulfilled in all patients) were prospectively enrolled between January 2017 and December 2019. Additionally, 30 sex-matched and age-matched healthy volunteers (14 males, 16 females, mean age 36.8 ± 2.3 years, range 19–64 years) were recruited. Exclusion criteria were age < 18 years, pregnancy, any contraindications for MRI, any risk factors for neuropathy such as alcoholism, diabetes, malignant or infectious diseases, a transition to secondary progressive multiple sclerosis, any therapy with steroids in the 8 weeks immediately prior to the MRN scans, and any previous exposure to neurotoxic drugs. By taking a detailed past medical history, any sensory or motor symptoms in the upper or lower extremities, any history of neuropathy, any previous spine surgery, and any chronic or malignant diseases were ruled out in all healthy volunteers.

### Clinical and Electrophysiological Examination

A past medical history was taken in all patients and all current and previous disease-modifying pharmacologic therapies were documented. Detailed clinical examination included scoring for the Expanded Disability Status Scale (EDSS) (R.D., A.V., M.K.-K., B.W.). Motor nerve conduction studies (NCS) assessed distal motor latencies (DML), compound muscle action potentials (CMAP), nerve conduction velocities (NCV), and F‑waves of the left tibial and peroneal nerves. Sensory nerve action potentials (SNAP) and NCVs were measured for the left sural nerve (M.W., G.S.). Skin temperature was controlled at a minimum of 32°.

### MRN Protocol

All participants underwent high-resolution MRN in a 3 T MR-scanner (Magnetom PRISMA, Siemens Healthineers, Erlangen, Germany) by using a 15-channel transmit-receive extremity coil (INVIVO, Gainesville, FL, USA) and the following sequence for T2-relaxometry:

Axial 2D dual echo turbo-spin-echo sequence with spectral fat saturation with four continuous imaging slabs at the left leg. Slab 1: proximal to mid-thigh; slab 2: mid to distal thigh with alignment of the distal edge with the tibiofemoral joint space; slab 3: proximal to mid lower leg with alignment of the proximal edge with the tibiofemoral joint space; slab 4: mid to distal lower leg with alignment of the distal edge with the tibiotalar joint space. One additional slab (slab 5) was acquired at the right leg covering the mid to distal thigh (equal position as slab 2 on the left side). Sequence parameters were: repetition time 5860 ms, short echo time (TE_1_) 14 ms, long TE (TE_2_) 86 ms, field of view 170 × 170 mm^2^, matrix size 512 × 512, slice thickness 3.5 mm, interslice gap 0.35 mm, voxel size 0.3 × 0.3 × 3.5 mm^3^, flip angle 180°, 35 slices, acquisition time per slab 8:25 min.

The net acquisition time for this protocol including survey scans was 44:39 min. Patient and coil repositioning required additional time, resulting in a total examination time of approximately 60 min per participant.

### Image Postprocessing and Statistical Analysis

All MRN images were pseudonymized, and subsequently analyzed with FSL software [17]. The tibial fascicles within the left sciatic nerve and their subsequent continuation as tibial nerve were manually segmented on a total of 140 axial imaging slices per participant from the proximal thigh to the tibiotalar joint space (imaging slabs 1–4) by one investigator blinded to clinical data (A.P.). The epineurium served as an easily visible segmentation border. To exclude relevant side differences, additional segmentation of the tibial fascicles within the right sciatic nerve was performed on 35 axial slices from right mid to distal thigh level (imaging slab 5). This anatomical level was chosen for side comparisons, as previous MRN studies in different polyneuropathies (PNP) as well as preliminary analyses in RRMS detected nerve lesions predominantly in this region [18–21].

### Quantitative and Semiquantitative Signal-based and Signal-independent Analyses

Signal quantification was performed by calculating the microstructural MRN markers, T2_app_ and ρ, according to the two following formulas by using data from the dual echo relaxometry sequence with TE_1_ set at 14 ms and TE_2_ set at 86 ms. This sequence has been shown to provide equally robust and reliable T2-relaxometry data when calculating T2_app_ and ρ as gold standard multi-echo sequences [22].

$$T2_{\mathrm{app}}=\frac{TE_{2}-TE_{1}}{\mathrm{ln}(SI(TE_{1})/SI(TE_{2}))}$$

$$\rho =\frac{SI\left(TE_{1}\right)}{\exp (-TE_{1}/T2_{\mathrm{app}})}$$

Mean values of tibial nerve T2_app_ and ρ were calculated per slice position and participant. We refrained from calculating ρ and T2_app_ on a pixel-by-pixel basis, as the high spatial resolution mandatory for MRN results in higher noise levels, which in turn lead to low accuracies of ρ and T2_app_ within single pixels. To decrease the influence of noise, we calculated ρ and T2_app_ from signal intensities determined in the nerve on each slice (region of interest approach).

Averaged mean values of tibial nerve T2_app_ and ρ were then compared between RRMS and healthy controls, as well as between proximal (thigh; imaging slabs 1 and 2) and distal (lower leg; imaging slabs 3 and 4) segments of the tibial nerve along the left leg in order to determine the exact spatial distribution pattern of peripheral nerve lesions in RRMS. Additionally, mean values of tibial nerve T2_app_ and ρ were compared between the left and right mid to distal thigh.

In the same way, mean values for semiquantitative tibial nerve T2w signal were additionally calculated.

Nerve cross-sectional area (CSA) represents a signal-independent, pure morphometric, quantitative MRN marker. Mean CSA of the tibial nerve was recorded per slice position and participant analog to the methods described for signal-based analyses.

### Statistical Analysis

Statistical data analyses were performed with GraphPad Prism version 9.0.2 for Windows (GraphPad Software, San Diego, CA, USA; J.C.H.). The Mann-Whitney test was used to detect differences between (i) RRMS patients and controls, (ii) proximal and distal anatomical locations, and (iii) the right and left leg for all evaluated MRN parameters. Pearson’s correlation coefficients were calculated for further correlation analyses between MRN parameters and demographic (participant age, sex, height, weight, body mass index, BMI), clinical (EDSS, duration of symptoms) and electrophysiologic (tibial and peroneal NCV, DML, and CMAP, sural nerve NCV and SNAP) results. Statistical tests were two-tailed and an alpha level of significance was defined at *p* < 0.05. All results are documented as mean values ± standard error of the mean (SEM).

## Results

### Clinical and Electrophysiologic Data

All 35 RRMS patients fulfilled the revised 2017 McDonald criteria, [9] and with the exception of one therapy-naïve patient, all patients were treated with different disease-modifying drugs. Current or previous disease-modifying drugs included in alphabetic order: alemtuzumab, dimethyl fumarate, fingolimod, glatiramer acetate, interferon beta 1a and 1b, natalizumab, and teriflunomide. Duration of symptoms at the time of MRN acquisition was between 5 months and 27.3 years with 10 of 35 patients being diagnosed within the last 3 years. Besides minor unspecific abnormalities in 12 RRMS patients, electrophysiologic examination results were all in physiologic ranges excluding the presence of a (poly)neuropathy. There were no group differences between RRMS patients and healthy controls in terms of age, weight, and height (Table 1). Detailed demographic, clinical, and electrophysiologic data are summarized in Table 1.

**Table 1:** Demographic, clinical, and electrophysiologic data

Parameter	RRMS patients	Controls	P value
Age (years)	37.7 ± 2.2	36.8 ± 2.3	0.72
Sex (M/F)	12/23	14/16	N/A
Body weight (kg)	75.7 ± 2.8	75.1 ± 2.4	0.57
Height (cm)	173.8 ± 1.7	174.3 ± 1.6	0.85
Duration of symptoms (months)	97.6 ± 15.3	N/A	N/A
EDSS (0–10 points)	1.6 ± 0.3	N/A	N/A
Tibial nerve CMAP (mV)	21.3 ± 1.3	N/A	N/A
Tibial nerve NCV (m/s)	50.5 ± 0.9	N/A	N/A
Tibial nerve F‑wave (ms)	49.4 ± 1.2	N/A	N/A
Tibial nerve DML (ms)	3.5 ± 0.1	N/A	N/A
Peroneal nerve CMAP (mV)	7.6 ± 0.6	N/A	N/A
Peroneal nerve NCV (m/s)	49.2 ± 1.0	N/A	N/A
Peroneal nerve F‑wave (ms)	47.8 ± 1.1	N/A	N/A
Peroneal nerve DML (ms)	4.0 ± 0.1	N/A	N/A
Sural nerve SNAP (µV)	15.2 ± 1.6	N/A	N/A
Sural nerve NCV (m/s)	52.3 ± 1.7	N/A	N/A

Results are presented as mean values ± SEM

*CMAP* compound muscle action potential, *DML* distal motor latency, *EDSS* Expanded Disability Status Scale, *N/A* not applicable, *NCV* nerve conduction velocity, *RRMS* relapsing-remitting multiple sclerosis, *SNAP* sensory nerve action potential

### Quantitative and Semiquantitative Signal-based and Signal-independent Analyses

#### Apparent T_2_-Relaxation Time (T2_app_)

**Fig. 1:**
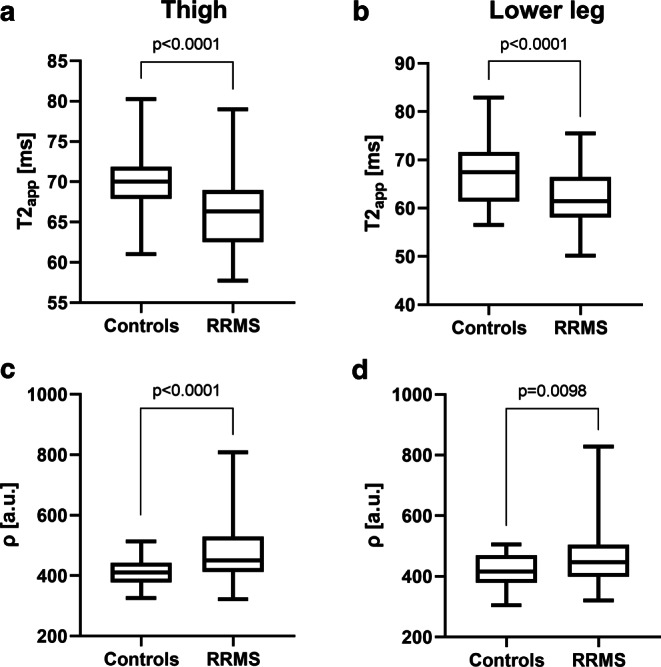
Quantitative microstructural MRN markers. Nerve T2_app_ (**a**, **b**) and nerve ρ (**c**, **d**) mean values at the thigh (**a**, **c**) and at the lower leg (**b**, **d**) were plotted separately for controls and RRMS in a box and whisker plot. Both microstructural markers differentiated well between RRMS patients and healthy controls when measured at the thigh and also at the lower leg. Significant differences are indicated by respective *p* values. *a.u.* arbitrary units, *ρ* proton spin density, *RRMS* relapsing-remitting multiple sclerosis, *T2*_*app*_ apparent T2-relaxation time

Tibial nerve T2_app_ was lower in RRMS at the thigh (66.1 ± 0.6 ms) and at the lower leg (62.1 ± 0.7 ms) compared to controls (thigh: 69.9 ± 0.6 ms, *p* < 0.0001; lower leg: 67.5 ± 1.0 ms, *p* < 0.0001; Fig. 1a,b). A proximal-to-distal gradient in tibial nerve T2_app_ from the thigh to the lower leg was identified in RRMS (*p* < 0.0001) and controls (*p* = 0.0267). Differences between tibial nerve T2_app_ at the left versus the right mid to distal thigh were not observed in RRMS (left 65.8 ± 0.7 ms versus right 65.9 ± 0.8 ms, *p* = 0.91) or controls (left 69.2 ± 0.9 ms versus right 69.4 ± 0.8 ms, *p* = 0.92). When separating the RRMS group into patients who were diagnosed less than 3 years ago (RRMS < 3 years) and those diagnosed more than 3 years (RRMS > 3 years) ago, ANOVA revealed group differences at the thigh (*p* < 0.0001, F = 10.56) and at the lower leg (*p* < 0.0001, F = 10.17). In detail, T2_app_ was lower in RRMS > 3 years (thigh: 66.3 ± 0.7 ms, *p* = 0.0004; lower leg: 62.3 ± 0.8 ms, *p* = 0.0003) and in RRMS < 3 years (thigh: 65.6 ms, *p* = 0.0012; lower leg: 61.6 ms, *p* = 0.0029) compared to controls, while differences between RRMS > 3 y and RRMS < 3 years did not exist (thigh: *p* = 0.86; lower leg: *p* = 0.91).

Tibial nerve T2_app_ at the thigh showed an inverse correlation with RRMS patient age (*p* = 0.0101, r = −0.3351) and the EDSS (*p* = 0.0209, r = −0.3080), but not with any other demographic, or clinical parameters; however, positive correlations were found between proximal tibial nerve T2_app_ and tibial nerve CMAPs (*p* = 0.0142, r = 0.3204) and NCVs (*p* = 0.0315, r = 0.2827). No further correlations were found between proximal or distal tibial nerve T2_app_ and any other demographic, clinical, or electrophysiologic parameters.

#### Proton Spin Density (ρ)

Tibial nerve ρ was higher in RRMS patients at the thigh (473.2 ± 12.1 a.u.) and at the lower leg (461.2 ± 11.4 a.u.) compared to controls (thigh: 410.1 ± 6.1 a.u., *p* < 0.0001; lower leg: 418.0 ± 7.5 a.u., *p* = 0.0098; Fig. 1c,d). A proximal-to-distal gradient between tibial nerve ρ at thigh and at lower leg level was not present in RRMS (*p* = 0.31) or controls (*p* = 0.43). Likewise, there were no side differences in tibial nerve ρ in RRMS (left 482.0 ± 18.4 a.u. versus right 456.3 ± 10.1 a.u., *p* = 0.39) or controls (left 412.9 ± 7.1 versus right 413.8 ± 7.0; *p* = 0.99). Group differences were identified between RRMS < 3 years vs. RRMS > 3 years vs. controls at the thigh (*p* < 0.0001, F = 11.71) and at the lower leg (*p* = 0.0011, F = 7.278). Post hoc analyses identified differences between RRMS > 3 years (thigh: 483.8 ± 14.2 a.u.; lower leg: 473.9 ± 14.0 a.u.) and controls (thigh: *p* < 0.0001; lower leg: *p* = 0.0009), but not between RRMS < 3 years (thigh: 442.8 ± 22.1 a.u.; lower leg: 427.9 ± 16.2 a.u.) and controls (thigh: *p* = 0.29; lower leg: *p* = 0.88) or between RRMS > 3 years and RRMS < 3 years (thigh: *p* = 0.15; lower leg: *p* = 0.08).

The ρ of the tibial nerve at the thigh or at the lower leg did not correlate with any demographic, clinical, or electrophysiologic parameters.

#### T2w Signal

**Fig. 2:**
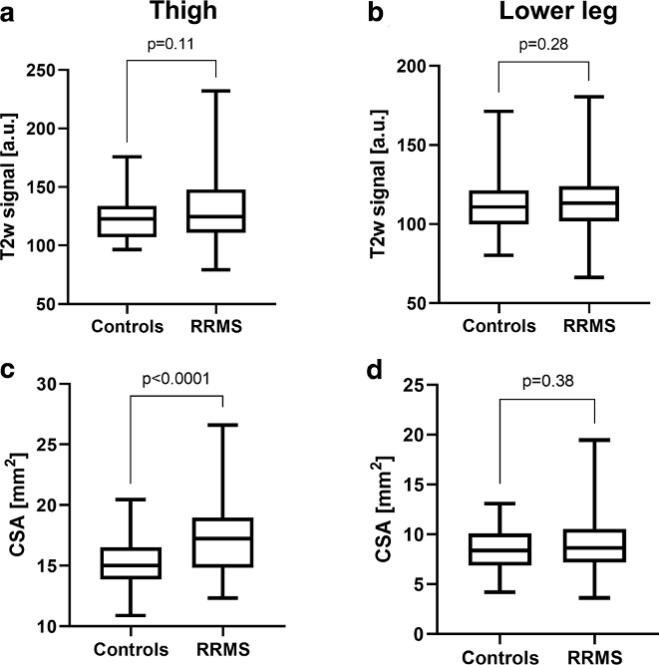
Semi-quantitative T2w signal and quantitative morphometric MRN markers. Nerve T2w signal (**a**, **b**) and nerve CSA (**c**, **d**) mean values at the thigh (**a**, **c**) and at the lower leg (**b**, **d**) were plotted separately for controls and RRMS in a box and whisker plot. While nerve T2w signal did not separate between RRMS patients and healthy controls, nerve CSA was higher in RRMS than in controls, but only when measured at the thigh and not at the lower leg. Significant differences are indicated by respective *p* values. *CSA* cross-sectional area, *RRMS* relapsing-remitting multiple sclerosis

**Fig. 3:**
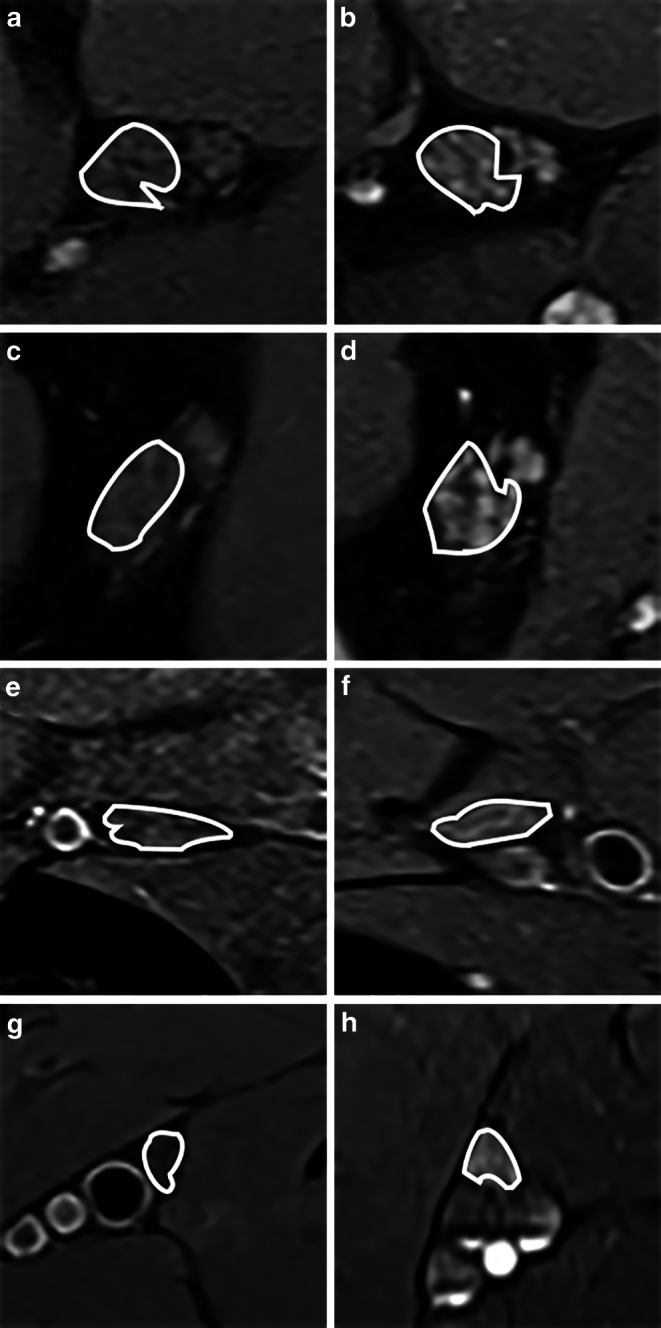
MRN source images. Representative magnetic resonance neurography (MRN) images (axial dual-echo turbo spin echo relaxometry sequences with spectral fat saturation) at the left proximal thigh (**a**, **b**), distal thigh (**c**, **d**), proximal lower leg (**e**, **f**), and distal lower leg (**g**, **h**) are shown at equal slice positions in a healthy control (*left:* **a**, **c**, **e**, **g**) and a patient with relapsing-remitting multiple sclerosis (*right:* **b**, **d**, **f**, **h**). Details show the segmented tibial fascicles within the sciatic nerve (**a**–**d**) and their distal continuation as tibial nerve (**e**–**h**). Note the diffuse, heterogeneous, and hyperintense lesion distribution in RRMS compared to the control. In RRMS, nerve CSA was also higher at the proximal and distal thigh, but not at the lower leg

Additional evaluations of tibial nerve T2w signal revealed no differences between RRMS and controls at thigh level (RRMS 132.1 ± 5.9 a.u. versus controls 122.6 ± 2.9 a.u., *p* = 0.11; Fig. 2a) or at the lower leg (RRMS 115.5 ± 2.8 a.u. versus controls 111.3 ± 2.6 a.u., *p* = 0.28; Fig. 2b). A proximal-to-distal gradient in tibial nerve T2w signal was detected in RRMS patients (*p* = 0.0008), but not in controls (*p* = 0.087). Differences in tibial nerve T2 signal between the left and right mid to distal thigh were not detected (RRMS: left 133.6 ± 5.8 a.u. versus right 129.6 ± 3.9 a.u., *p* = 0.76; controls: left 123.4 ± 4.5 a.u. versus right 120.6 ± 3.0 a.u., *p* = 0.74). Upon visual evaluation, nerve lesion distribution in RRMS was disseminated and heterogeneous with slightly hyperintense fascicles being located next to normointense appearing nerve fascicles on nerve cross-sections (Fig. 3).

Tibial nerve T2w signal inversely correlated with male sex (thigh: *p* = 0.0005, r = −0.4460; lower leg: *p* = 0.0012, r = −0.4045), and tibial nerve F‑waves (thigh: *p* = 0.032, r = −0.2867; lower leg: *p* = 0.0067, r = −0.3495), while correlating positively with tibial nerve CMAPs (thigh: *p* = 0.0094, r = 0.3385; lower leg: *p* = 0.0128, r = 0.3169) and NCVs (thigh: *p* = 0.0188, r = 0.3076; lower leg: *p* = 0.0039, r = 0.3647). Tibial nerve T2 signal at the lower leg did not correlate with any demographic, clinical, or electrophysiologic parameters.

#### Cross-sectional Area (CSA)

Tibial nerve CSA at the thigh was slightly increased in RRMS (17.3 ± 0.4 mm^2^) versus controls (15.1 ± 0.3 mm^2^, *p* < 0.0001; Fig. 2c), while no such differences existed at the lower leg (RRMS 9.1 ± 0.4 mm^2^ versus controls 8.5 ± 0.3 mm^2^, *p* = 0.38; Fig. 2d). As expected and due to the physiologic anatomical course, tibial nerve CSA was higher at the thigh than at the lower leg in RRMS and controls (each *p* < 0.0001). Analysis of tibial nerve CSA at mid to distal thigh level revealed no differences between the left and right side (RRMS: left 66.6 ± 2.2 mm^2^ versus right 65.4 ± 1.6 mm^2^, *p* = 0.96; controls: left 58.4 ± 1.4 mm^2^ versus right 58.8 ± 1.6 mm^2^, *p* = 0.91).

Tibial nerve CSA at the thigh correlated positively with patient weight (*p* = 0.0076, r = 0.3530), male sex (*p* = 0.0151, r = 0.3234), the EDSS (*p* = 0.0418, r = 0.2781) and disease duration (*p* = 0.0168, r = 0.3183). Inverse correlations were detected between tibial nerve CSA at thigh level and tibial nerve CMAPs (*p* = 0.0016, r = −0.4125), and sural nerve SNAPs (*p* = 0.0173, r = −0.3171) and NCVs (*p* = 0.0337, r = −0.2843). No correlations with any demographic, clinical, or electrophysiologic parameters were found for tibial nerve CSA at the lower leg.

## Discussion

Demyelination in MS is traditionally regarded as an autoimmune disease exclusively affecting the CNS. Even though several histopathologic studies detected areas of additional demyelination in the peripheral nerves of MS patients, these findings were attributed to concomitant neuropathies caused by malnutrition or anemia, rather than to MS itself [10–12, 23, 24]. Electrophysiologic studies were inconclusive and showed an inhomogeneous picture of peripheral nerve electrophysiologic properties in MS [13, 25]. A recent pilot study applying MRN with high structural resolution, conducted by Jende et al., was the first to reliably demonstrate peripheral nerve involvement in an unselected cohort of MS patients by directly visualizing peripheral nerve lesions in vivo [15]. That study was mainly based on the proximal-to-distal assessment of nerve T2w signal at the lower extremities, a MRN parameter that cannot be directly quantified, but can be influenced by external factors, such as field inhomogeneities, radiofrequency excitation field inhomogeneity, nonuniform receiver coil sensitivity, or different signal attenuation for the imaged slabs [16]. Additional morphometric, signal-independent information was provided by measuring proximal-to-distal alterations of nerve CSA; however, a major limitation of the previous study was that more important quantitative microstructural data derived from T2 relaxometry, necessary to understand the pathomorphologic origin behind the occurrence of PNS lesions in MS, was only gathered at a small anatomical section at the distal thigh [15].

Here, we present a comprehensive characterization and quantification of lower extremity peripheral nerve involvement from the proximal thigh to the distal lower leg in a cohort of clinically, and electrophysiologically well-examined patients exclusively with RRMS. Our results show (i) that peripheral nerve lesions in RRMS are characterized by a decrease in T2_app_ and an increase in ρ and CSA, while no differences were observed for T2w signal, and (ii) that the microstructural quantitative MRN marker T2_app_ and ρ are altered at the thigh and the lower leg with proximal predominance.

T2_app_ and ρ are quantitative MRN markers that provide supplementary information on the integrity and macromolecular composition of nerve tissue by reflecting changes in the biochemical microstructure [26–31]. Initially, both markers were utilized for imaging of the CNS, before they were successfully applied in the PNS [18]. In recent years, T2_app_ and ρ were increasingly used to quantify nerve damage in a multitude of different diffuse neuropathies, in which distinct directions of change in T2_app_ and ρ (either combined or opposing decrease and/or increase in T2_app_ and ρ) were identified representing the underlying disease entity. In detail, PNPs of different etiology, such as hereditary transthyretin (ATTRv) amyloidosis, systemic light chain amyloidosis, diabetic, and alcoholic neuropathy were characterized by an early increase in ρ, indicating demyelination, while T2_app_ was additionally increased in more advanced stages [18–21, 32]. Furthermore, ρ detected nerve damage in clinically and electrophysiologically completely asymptomatic carriers of the variant transthyretin gene or in alcohol-dependent patients without clinically overt PNP [18, 21, 32]. Nerve damage in the neurodegenerative motor neuron disease, 5q-linked spinal muscular atrophy (SMA), was characterized by an increase in T2_app_ and a decrease in ρ [33], which can be explained by the decay of lower motor neurons and the subsequent axonal loss accounting for the predominant pathomorphologic mechanism in SMA. In RRMS, as an example of a demyelinating disease, nerve lesions were characterized by a decrease in T2_app_ and an increase in ρ, a unique alteration among the neuropathies investigated with quantitative MRN so far.

Since MRN was first developed three decades ago, an increase in nerve T2w signal has become the most established, yet unspecific MRN criterion to detect nerve damage, especially in traumatic nerve injury or entrapment neuropathies [34–41]. A prolonged T2_app_, caused by an increase in the number of free water protons in the extracellular compartment, was suspected as the main factor leading to a T2w signal increase [42–44]; however, in our RRMS cohort, T2w signal along the sciatic and tibial nerves was not significantly altered, yet a multitude of nerve fascicles appeared to be hyperintense upon visual inspection of the acquired T2 relaxometry sequences (Fig. 3). The observed decrease in T2_app_ might appear contradictory, however, the formula calculating the T2 decay (S (TE) = ρ * exp (−TE / T2_app_); S = signal, TE = echo time) demonstrates that a combined increase in T2_app_ and ρ, an increase of one parameter with constancy of the other parameter, or an increase of one parameter outweighing the decrease of the other parameter, can lead to an increased T2w signal. In RRMS, the visually observed T2w hyperintensity in many nerve fascicles was caused by an increase in ρ that was more pronounced than the decrease in T2_app_.

Our study results confirm the findings of a previously published proof-of-concept study [15] in an entirely new patient cohort of solely RRMS patients and show that peripheral nerve lesions in RRMS are characterized by an increase in ρ and a decrease in T2_app_. Alterations in the two microstructural MRN markers can be similarly observed at proximal (thigh) and distal (lower leg) peripheral nerves; however, a strong proximal-to-distal gradient was detected for T2_app_ but not for ρ. This finding, further supported by the visual impression of a diffuse and heterogeneous appearance of nerve lesions, indicates a disseminated, yet proximally predominant distribution of peripheral nerve lesions in RRMS. Importantly, the observed proximal-to-distal distribution pattern supports the hypothesis that peripheral nerve involvement is directly associated with MS and not caused by a neuropathy of different origin. If peripheral nerve lesions were to occur secondary to more proximally located CNS lesions (for example due to Wallerian degeneration), peripheral nerve lesions would appear rather continuous and segmental [45, 46]. Moreover, a previously published study found an inverse correlation between spinal cord and sciatic nerve T2w-hyperintense lesions [15]. A concomitant PNP can also be excluded, as electrophysiologic examination results were in physiologic ranges, and the nerve lesion distribution as detected by MRN would be expected to appear with a proximal-to-distal gradient in ρ [18–20, 47]. In our RRMS cohort, the increase in ρ occurring without a gradient along the proximal and distal tibial nerve, may reflect a potential co-demyelination of peripheral nerves. Support for this hypothesis comes from a histopathologic CNS study where areas of increased ρ in the brain and spinal cord of deceased MS patients correlated with areas of demyelination [48]. While it is known that alterations of ρ are induced by a changes in the macromolecular composition of nerve tissue, the factors that directly contribute to a ρ increase in MS are not fully understood. One possible explanation is a disruption of the endovascular or rather blood nerve barrier caused by inflammatory processes and an impairment of the lipid-rich myelin sheath, subsequently leading to an increased leakage of plasma proteins [49]. As a result, water molecules might increasingly bind to macromolecules, decreasing the amount of free water protons, which in turn would additionally explain the decrease in T2_app_. Moreover, the observed decrease in T2_app_ points against an endoneural edema as an important contributor of peripheral nerve lesions in RRMS. Here, ongoing studies evaluating magnetization transfer contrast imaging might contribute to a better understanding of interactions between free water molecules and protons bound to macromolecules in the future.

Despite the PNS involvement in RRMS, patients in our cohort remained without overt clinical or electrophysiologic signs of a peripheral neuropathy; however, besides ρ, all analyzed MRN markers correlated with certain electrophysiologic parameters or the EDSS, indicating that MRN findings represent alterations in nerve conductivity on a subclinical level. Moreover, the observed inverse correlation of T2_app_ and the positive correlation of CSA with the EDSS might reflect a functional impairment of the PNS in MS, potentially contributing to the overall clinical symptom presentation; a hypothesis that might explain the often inexplicable gap between the severity of clinical symptoms and a comparably low burden of CNS lesions [50, 51]. Similar findings have been observed in asymptomatic carriers of the variant transthyretin gene, where MRN preceded the clinical and electrophysiologic disease onset [18, 32]. Even though the pathomechanism leading to the occurrence of PNS lesions in RRMS is unclear, several studies hypothesized that immune cells or antibodies directed against epitopes that are common to the CNS and PNS might play an important role. As an example, connexins as parts of gap junctions between myelinating cells, were assumed to be involved in a combined PNS and CNS demyelination [23, 24, 52]. Other studies focusing on the transition zone between the central and the peripheral myelin of the trigeminal nerve implied that specific proteins, such as connexin 32 and myelin basic protein, are targeted by inflammatory T cells in MS [24, 53].

Our study is limited by its cross-sectional design that does not allow an interpretation regarding the temporal evolution of peripheral nerve lesions in MS. Future studies investigating the PNS in newly diagnosed MS patients or in patients with clinically or radiologically isolated syndromes would be desirable to study early peripheral nerve involvement. Additional histopathologic studies in patients with RRMS would be beneficial to prove or exclude whether alterations in T2_app_ and ρ represent subclinical nerve damage. Furthermore, an additional acquisition and analysis of cerebral and spinal MRI in RRMS patients might clarify whether a combined demyelination of the CNS and PNS occurs or PNS lesions precede CNS manifestation. Evaluation of quantitative MRN markers may be difficult to perform in clinical settings as they require a time-consuming manual segmentation; however, identification of peripheral nerve involvement in RRMS seem to be sufficiently possible based on visual inspection by an experienced radiologist [15].

Our study provides first comprehensive data on the in vivo characterization and proximal-to-distal spatial distribution of peripheral nerve involvement in patients with RRMS. Peripheral nerve lesions in RRMS are characterized by a decrease in T2_app_, and an increase in ρ. While significant differences in quantitative markers could be similarly observed at the thigh and the lower leg, a strong proximal-to-distal gradient was detected for T2_app_ but not for ρ, indicating a disseminated, yet proximal predominance of peripheral nerve lesions. These findings may further contribute to the pathophysiologic understanding and relevance of PNS involvement in RRMS.
